# 22 A Burn Specialty Learning Experience: The Burn Center Symposium

**DOI:** 10.1093/jbcr/irae036.022

**Published:** 2024-04-17

**Authors:** Michael G Gleason, Sarah Shingleton, Maria L Serio-Melvin, Maria Gonzalez, Cullen Boyd, Patricia Colston, Kristine N Chafin

**Affiliations:** USAISR, San Antonio, Texas; US Army Institute of Surgical Research, Cibolo, Texas; USAISR, San Antonio, Texas; US Army Institute of Surgical Research, Cibolo, Texas; USAISR, San Antonio, Texas; US Army Institute of Surgical Research, Cibolo, Texas; USAISR, San Antonio, Texas; US Army Institute of Surgical Research, Cibolo, Texas; USAISR, San Antonio, Texas; US Army Institute of Surgical Research, Cibolo, Texas; USAISR, San Antonio, Texas; US Army Institute of Surgical Research, Cibolo, Texas; USAISR, San Antonio, Texas; US Army Institute of Surgical Research, Cibolo, Texas

## Abstract

**Introduction:**

The Covid-19 pandemic disrupted educational activities in our burn center and across the nation. Findings from organizational climate surveys and the American Burn Association (ABA) verification process suggested that continuing education and professional development opportunities required improvement. Clinical Nurse Specialists (CNS) collaborated with the burn center education department (BCED) to revitalize an outdated Burn Center Symposium (BCS) which was used to provide training for novice and seasoned interprofessional staff. The BCS was aligned with the ABA Burn Nurse Competency Initiative (BNCI) to provide a quarterly learning opportunity for all staff and prepare our nurses for the Certified Burn Registered Nurse (CBRN) Exam.

**Methods:**

CNSs and the BCED cross-walked the current BCS materials to verify alignment with the BNCI domains, national accreditation, and department requirements. The team then used BNCI essential performance criteria as a framework to create lesson outlines which were used by an interdisciplinary team to update lesson plans. Additionally, burn care simulations were created to practice emergency response, respiratory therapy, and rehabilitation scenarios. Multiple choice exam and Likert surveys were created to evaluate knowledge and quality. The BCS was offered to all disciplines in the burn center; descriptive statistics were used.

**Results:**

Two iterations of the BCS occurred in February (F) and July (J) 2023. F

**results:**

N=19 attendees, 52% < 3 years of burn experience, a 19% increase in exam scores occurred between an average of 56% presurvey and 75% postsurvey. J

**results:**

N=36, 90% had < 3 years of burn experience, a 15% increase in exam scores occurred between an average of 69% presurvey and 84% postsurvey. For both iterations, over 80% of the staff agreed or strongly agreed positively with course quality.

**Conclusions:**

The results suggest that using the BNCI domains as a framework for our BCS improved knowledge of both novice and seasoned burn nurses, as well as other members of the inter-professional team.

**Applicability of Research to Practice:**

Using the evidenced based BNCI domains as a framework for our inter-professional Burn Center Symposium improved knowledge and was the first step to prepare our nurses for the CBRN.

